# British Society of Breast Radiology: Bristol, UK. 3-5 November 2019

**DOI:** 10.1186/s13058-019-1225-x

**Published:** 2019-12-06

## Presentation Type: Oral

### 0013 Pre-operative prediction of margin requirement following a core biopsy suggestive of a phyllodes tumour.

#### Valentine Mberu^1^, E. Jane Macaskill^2^, Colin Purdie^2^, Andrew Evans^1^

##### ^1^University of Dundee, Dundee, United Kingdom; ^2^Ninewells Hospital, NHS Tayside, Dundee, United Kingdom

###### **Correspondence**: Valentine Mberu

**Background:** Definitive diagnosis of a phyllodes tumour can only be done after excision of the lesion. However, malignant and borderline phyllodes require resection with a margin while benign phyllodes and fibroadenomas do not. Pre-operative prediction of the need for a margin will be advantageous.

**Methods:** 31 lesions with a core biopsy suggestive of a phyllodes tumour were identified. Mammographic, ultrasound (US), and demographic data (age and source) were assessed while blinded to surgical outcomes. The features of lesions requiring a margin and those that did not were compared. Statistical analysis used Chi-square Fisher’s exact test and ROC curves.

**Results:** Of 31 assessed lesions, 13 required a margin and 18 did not. There were 6 screening-detected lesions, which were benign. Features found more frequently in those requiring a margin were poorly-defined margin on mammography [7/9 (78%) vs 4/13 (31%) p =0.04]; on ultrasound, irregular shape [8/13 (62%) vs 3/18 (17%) p= 0.01], microlobulations [7/13 (54%) vs 3/18 (17%) p = 0.028], mixed echogenicity [9/13 (69%) vs 1/18 (6%) p = 0.0002], echogenic clefts [6/13 (46%) vs 1/18 (6%) p = 0.007), BIRADS score > 3 [11/13 (85%) vs 9/18 (50%) p=0.047], distal enhancement [9/11 (82%) vs 6/18 (33%) p=0.01], ultrasound size and stiffness at shear-wave elastography, were also predictors: area under the curve (AUC) 0.76, p=0.003 and AUC 0.71, p=0.026 respectively.

**Conclusion(s):** We have identified pre-operative features which can be used to guide surgical choice of margin when excising lesions with a core biopsy suggestive of a phyllodes tumour.

### 0015 Data from 11.3 million screens in the NHSBSP. Is there an inverse relationship between grade and mammographic sensitivity?

#### Rosalind Given-Wilson^1^, Matthew Wallis^2^, Roger Blanks^3^

##### ^1^St Georges Hospital, London, United Kingdom; ^2^Addenbrookes Hospital, Cambridge, United Kingdom; ^3^Oxford University, Oxford, United Kingdom

###### **Correspondence:** Rosalind Given-Wilson

**Purpose:** There is a 20% mortality reduction in women invited to mammographic screening. Much of this is due to the detection of high grade invasive cancers. We examined sensitivity of detection of these cancers in the English breast screening programme (NHSBSP).

**Materials and methods:** This study examined data from national returns from the NHSBSP for 7 years, 2009/2010 to 2015/2016. Information on size and grade of invasive cancers was collected for prevalent (age 45-52) and routine repeat incident (age 53-70) screens.

**Results:** Data was analysed from 11.3 million screens when 67,681 invasive cancers were diagnosed. Overall 29% were grade 1, 52% grade 2 and 18% grade 3 at the prevalent screen. The lowest detection rates were seen for small grade 3 cancers ( under 15 mm) and large grade 1 cancers ( over 15mm). We estimate the sensitivity for grade 3 cancers to be around 50% , and grade 2 cancers about 62% of that for grade 1 cancers.

**Conclusions:** Data from the Swedish two counties study (STCS) showed the importance of detecting small grade 3 cancers in reducing mortality. Grade 3 cancers are over-represented in studies of interval cancers. Our data suggests relatively low sensitivity for small grade 3 cancers in the NHSBSP although similar to the STCS. This may be related to fast doubling time for high grade cancers and poor mammographic visibility. Future technological development in mammographic screening should focus on improving detection of high grade cancers.

### 0025 Comparison of margin visibility for masses on digital breast tomosynthesis (DBT) and 2D digital mammography (2DMM); can it help reduce recall and benign biopsy rate in the screening setting?

#### Nyla Khan, Linda Metaxa, Tamara Suaris

##### St Bartholomew's Hospital, Barts and The Royal London NHS Trust, London, United Kingdom

###### **Correspondence:** Nyla Khan

Sensitivity of 2DMM is limited which can cause uncertain evaluation of soft tissue masses; resulting in higher recall rates. This study compares margin visibility of masses on DBT and 2DMM to determine whether DBT can help predict benign lesions based on margin visibility, reducing recall and biopsy rate.

Retrospective study included women who were recalled from screening between 2017-2018 for benign looking lesions who underwent DBT and biopsy. Reader blind to biopsy results analyzed DBT and 2DMM images. Biopsy necessity was recorded as well as margin visibility which was categorised according to greatest percentage seen: Category I (0-25%) = border of the total margin visible, Category II= 26-50%, Category III= 51-75%, Category IV= 76-100%.

DBT identified 128 lesions as compared to 126 on 2DMM. Of 128 lesions; 12 malignant and 116 benign. Benign lesions demonstrated higher margin visibility on DBT, with 104 classified in Category III/IV compared with only 24 in the same category on 2DMM. There was a high degree of correlation between low margin visibility and risk of malignancy on DBT; with 10/12 malignant lesions in Category I/II, compared with 2/12 in Category III/IV. One B4 lesion with a Category IV margin was identified as a rare low-grade mucoepidermoid carcinoma with DCIS in situ. Inclusion of DBT to 2DMM would have reduced the number of biopsies by 66%.

DBT results in greater visualization of circumscribed masses; improved analysis of margin detail aiding in better prediction of both benign and malignant lesions, and reduction in the benign biopsy rate.

### 0070 Radio-wave image guided biopsy needle for breast diagnostic

#### Louis Tsui^1^, Peter Brady^2^, Mark Beaumont^1^

##### ^1^Micrima Ltd, Bristol, United Kingdom; ^2^Covensis, Farndon, United Kingdom

###### **Correspondence:** Louis Tsui

Electromagnetic radio-wave imaging is a new modality for medical diagnosis applications. MARIA® (Micrima Limited, Bristol UK) is a commercially available radio-wave breast imaging system that utilises the ability to detect the contrast in the dielectric properties between different tissue types. Radio-wave is non-ionising, and MARIA® requires no breast compression. Clinical trials [1] to date has shown evidence in identifying regions of interest requiring further diagnosis, one of which can be biopsy. This work presents the feasibility of how MARIA® could be used to first identify the area of investigation, provide a biopsy needle, and then guide it for biopsy examination.

A lesion mimicking phantom was placed within the image domain of MARIA®’s hemispherical array which compromises of 60 radio-wave antennas. The antenna closest to the identified phantom lesion location was then used to allow insertion of a biopsy needle. Further radio-wave signal was acquired as the needle was being inserted closer to the target, this provide a closed-loop feedback continuous guidance control. Figure 1 summarises the result.

This is the first presentation of any experimental result for a needle biopsy using radio-wave imagining and guidance. With further technical development, a patient breast scan and subsequence biopsy examination could be conducted over a single diagnostic session using MARIA® system.

[1] Share, M et al. (2019). “MARIA® M5: A multicentre clinical study to evaluate the ability of the Micrima radio-wave radar breast imaging system (MARIA®) to detect lesions in the symptomatic breast”, European Journal of Radiology, July 2019, Volume 116, Pages 61-67.

### 0074 Can real-life screening performance be predicted by an individual’s PERFORMS data?

#### Yan Chen^1^, Jonathan James^2^, Eleanor Cornford^3^, Jacquie Jenkins^4^

##### ^1^University of Nottingham, Nottingham, United Kingdom; ^2^Nottingham Breast Institute, Nottingham, United Kingdom; ^3^Gloucestershire Hospitals NHS Foundation Trust, Cheltham, United Kingdom; ^4^Public Health England, Sheffield, United Kingdom

###### **Correspondence:** Yan Chen

Readers who report screening mammograms in the NHS Breast Screening Programme participate annually in the PERFORMS educational scheme. The real-life performance of all readers is recorded in the Breast Screening Information System (BSIS).  The real-life cancer detection rates and recall rates of 447 readers over a three year period were acquired from BSIS.  These were matched to the individuals ‘area under the curve’ (AUC) scores derived from ROC analysis of their PERFORMS test set results. Real-life performance data from BSIS is frequently presented in the form of scatter plots of cancer detection rate plotted against  recall rate. PERFORMS AUCs were found to vary significantly by the quadrant into which a participants real-life performance fell on the scatterplot. Screeners with high sensitivity and specificity in real-life, and those with high sensitivity and low specificity in real-life had significantly higher PERFORMS AUC scores than screeners with low sensitivity and specificity in real-life (ANOVA *F*(3, 443) = 5.61, *p*< .001, ω^2^= .03). In Pearson correlations, AUC exhibited a significant positive correlation with real-life cancer detection rates (r = +.19, n = 447, p < .0001, two tails), but not with real-life recall rates (r = -.06, n = 447, p = n.s., two tails).

PERFORMS ROC scores can differentiate readers whose real-life screening performance is poor in terms of sensitivity and specificity from readers whose real-life screening performance is characterised by high sensitivity.

### 0096 Masking Risk Index: an evaluation to guide supplemental imaging for breast screening.

#### Sarah Hickman, Maria Pantelidou, James Mainprize, Martin Yaffe, Yuan Huang, Richard Black, Fiona Gilbert

##### University of Cambridge, Cambridge, United Kingdom ^2^Sunnybrook Research Institute , Toronto , Canada

###### **Correspondence:** Sarah Hickman

The study assessed a Masking Risk Index tool to predict the likelihood of a larger cancer being found. The tool can then be applied to predict cases where larger cancers might be found at a subsequent screening round, thus identifying a sub-population that would benefit from supplemental imaging for the detection of cancer at an earlier round.

The Masking Risk Index tool was tested on the contralateral (disease-free) mammograms of women recalled from screening, aged 54 to 83, acquired from a single vendor between 2011 and 2013. 361 lesions (352 cases) from circumscribed and spiculated invasive cancer cases, diameters ranging from 2mm to 105mm, were included in the study; 208 of the lesions were <15mm. A masking index threshold >0.3 equals masked.

A linear mixed-effects model was used to investigate the association between masking index and lesion size, where hierarchical categories were treated as random effects. The model indicated that for each 0.1 increase in the masking index, the detectable lesion size was expected to rise by 5.6% (p=0.01). Using the chi-square test, where cut-off values from masking index and lesion size were 0.3 and 15mm respectively, conveyed that lesions with masking index ≥0.3 showed significantly higher frequencies of large tumour (p=0.03). This was further confirmed using a Mann-Whitney U-test (15.0 [11.0, 20.5] mm vs. 13.0 [9.0, 18.0] mm, p=0.03).

The use of decision algorithms such as the Masking Risk Index tool can ensure efficient selection of women who would benefit most from supplemental imaging while avoiding its overuse.

### 0089 Breast Cancer: Early diagnosis using materials immortalisation

#### Keith Rogers^1^, Iain Lyburn^2^, Sarah Gosling^1^, Nicholas Stone^3^, Nallala Krupakar^3^

##### ^1^Cranfield University, Swindon, United Kingdom ^2^Cobalt Health, Cheltenham, United Kingdom ^3^Exeter University, Exeter, United Kingdom

###### **Correspondence:** Keith Rogers

Radiographic mammary calcifications occur in approximately 40% of breast cancers and are often the sole feature to indicate tumour presence. Around 50% of breast biopsies performed for non-palpable mammographic abnormalities are to assess microcalcification. There is significant, growing, international academic interest in the nature of such pathological calcifications, which may be a feature of many types of lesion, including prostate, ovarian, uterine and thyroid, as well as breast cancer. The number of papers in Pubmed on apatite/hydroxyapatite calcifications in cancer has more than doubled in the last five years partly due to the growing recognition that calcifications can result from increased expression of bone matrix proteins in cancer cells and also due to evidence of a role in proliferation and migration of tumour cells.

The composition and material characteristics of calcifications remain poorly understood. A detailed understanding of physicochemistry / pathology relationships is required before translation to the clinic. There is also a growing recognition that the presence and physical form of calcifications is linked to prognosis, but calcification chemistry as a prognostic indicator remains entirely unexplored.

The current state of the art in terms of clinical significance of calcification chemistry will be presented and the potential for new diagnostic and prognostic markers explored.

### 0090

**WITHDRAWN**

## Presentation Type: Poster

### 0014 NHS Breast Screening Programme - Can we safely reduce our clinical recall rate?

#### Jennifer Birtchnell, Gillian Hutchison

##### The Nightingale Centre, Manchester, United Kingdom

###### **Correspondence:** Jennifer Birtchnell

Women attending for routine breast screening may be recalled for an assessment due to clinical signs or symptoms, to rule out mammographically occult disease. At recall, further imaging, clinical examination +/- needle sampling is performed. National standards: “To minimise the number of women screened who are referred for further tests”. Anecdotally clinical recalls are not felt to yield a high cancer detection rate.

Can the clinical signs described by ladies or the mammographers be dismissed without further assessment by

- Identifying the number of women clinically recalled to assessment over a 3-year round length
- Ascertaining the reason for clinical recall and result of assessment.
- Identifying the cancer detection rate in this population.

5,689 recalled to assessment - 173 (3%) for a clinical recall, 136 were lumps.

108 women were returned to routine screening with no abnormalities found, a further 63 discharged with benign findings.

10 core biopsies performed:

– two women diagnosed with a breast malignancy – in retrospect both had subtle signs on mammography.

The cancer detection rate in this group of women is considerably lower than the NHS BSP target of 3.6 per 1000. Minimising harm from screening is essential and includes unnecessary assessment. This work suggests that clinical recalls without additional mammographic abnormality do not yield a sufficiently high cancer detection rate to justify their recall. This could reduce numbers in assessment clinics staffed by a markedly dwindling workforce. Collaboration with neighbouring screening units is being pursued to evaluate this with larger numbers.

### 0017 Role of combined mammographic and MRI surveillance in patients with previous ‘mammographic occult’ breast cancer and other high risk patients.

#### David Purchase^1^, Matthew Wray^1^, Dr G J Bansal^2^

##### ^1^Cardiff University School of Medicine, Cardiff, Wales, United Kingdom; ^2^The Breast Centre, University Hospital Llandough, Cardiff and Vale University Health Board, Cardiff, United Kingdom

###### **Correspondence:** David Purchase

**Background:** To evaluate the role of combined MRI and mammogram follow up in patients with previous ‘mammographic occult’ breast cancer and other high-risk patients including those carrying genetic mutations.

**Methods:** Between 2011 and 2016, images of all patients undergoing routine surveillance following previous mammographically occult breast cancer and high- risk patients were evaluated. Most patients had both MRI and mammograms at intervals of 12- 18 months. Total number of recalls on both imaging modalities and the outcome of those recalls was recorded. Clinical details of all cancers were recorded, including the visibility on images and clinical presentation.

**Results:** There were a total of 508 images of 84 patients. There were 150 mammograms/MRI pairs and 136 lone MRI and 72 lone mammograms. The median age at start of follow up was 46 years (26- 75 years) and median number of images per patient was six. There was a total of 43 recalls in 34 patients. Recall rate was higher for MRI versus mammograms (10.8% versus 4.95%). MRI had low specificity 60.1% compared to mammograms (84.5%) due to high false positives but with similar sensitivity. 3 out of 5 cancers (60%) presented symptomatically.

**Conclusions:** MRI surveillance leads to higher recalls and false positives compared to mammograms in this specific subgroup of high-risk patients. 60% of cancers presented symptomatically, stressing the importance of remaining vigilant of breast symptoms despite imaging surveillance.

### 0026 Overview of breast manifestations of systemic disease

#### Sophia Tincey, Kesavan Nayagam, Dylan Tsukagoshi, Ros Crooks, Brian Holloway, Anmol Malhotra

##### Royal Free NHS Foundation Trust, London, United Kingdom

###### **Correspondence:** Sophia Tincey

The aim of this poster is to improve awareness and understanding of pathological breast manifestations of systemic diseases. Several systemic illnesses that can affect the breast will be discussed and we will summarise key clinical features and histopathology for each condition. Furthermore, we will discuss the imaging findings associated with each disease using multi-modality radiological examples.

The majority of breast pathology primarily focuses on breast cancer with little emphasis placed on how systemic illness can affect the breast; often giving rise to concern related to diagnosis and treatment. Women exhibiting breast manifestations of systematic disease may present with a variety of symptoms such as a mass, skin changes and focal pain, with or without mammographic abnormalities. After excluding primary breast malignancy or benign primary breast pathology, evaluation for systemic disease should be considered in these patients where diagnosis remains unclear.

Radiological features of systematic disease can mimic those of primary breast malignancy giving rise to diagnostic challenges, even if the underlying disease is known. Features of systematic disease may be apparent on mammography, ultrasound, CT and MRI, athough are often not well recognised. It is therefore important for radiologists to distinguish some of the key imaging features associated with these conditions when interpreting these modalities.

Breast cancer must always be strongly considered in the differential diagnosis for patients presenting with breast symptoms. However, despite the rarity of systemic disease involvement, radiologists should have a good overall understanding of associated breast features to aid in appropriate evaluation and treatment of patients.

### 0027 How reliable is contrast enhanced spectral mammography (CESM) in assessing the response to neo-adjuvant chemotherapy (NAC) in patients diagnosed with ductal breast carcinoma? Our experience in a DGH

#### Georgiana Zamfir, Ketki Khadtare, Mary Bourak, George Kousparos, Emily Daulton, Kirsten Stafford, Fiona Hearn, Philippa Skippage

##### Frimley Park Hospital, Frimley, United Kingdom

###### **Correspondence:** DR Georgiana Zamfir

**Background**: Contrast enhanced mammography (CESM) is an upcoming modality for breast cancer imaging which uses dual energy acquisition with contrast enhancement to provide vascular assessment and, hence, supplement the morphological information obtained from Full Field Digital Mammography (FFDM).

**Methods:** We looked back at our experience of CESM for assessment of post-chemotherapy response in ductal breast carcinoma from October 2017 to March 2019 and retrospectively reviewed imaging data for feasibility, accuracy, and technical problems. The diagnostic accuracy was correlated with that of ultrasound, which is the imaging mainstay for assessing tumour response in our unit​, alongside review of post-surgical pathology.

**Results**: We found that CESM proved to be a better tool as compared to ultrasound for assessing tumour response. Also, CESM has a potential to demonstrate microcalcifications on the unsubtracted images as opposed to MRI and ultrasound which are not reliable for assessment of microcalcifications. There remain some limitations to CESM such as contraindications to iodinated contrast media and hindering metallic artefacts from localiser clips.

**Conclusion**: We concluded that, although, there are some challenges for practical implementation, CESM is a better imaging alternative to ultrasound to evaluate tumor response in our unit. It also comes across as a feasible alternative to MRI, which is widely considered as the gold standard in this context, particularly due to better patient tolerance, lesser study times and cost effectiveness.

### 0028 Imaging evaluation of Paget’s disease of nipple- Are we doing enough?

#### Sau Lee Chang, Karen Gray

##### University Hospital Hairmyres, Glasgow, United Kingdom

###### **Correspondence:** Karen Gray

Paget’s disease is highly associated with underlying ductal carcinoma in-situ (DCIS). However, the diagnosis is often underestimated for radiographically occult DCIS. Surgeons find it difficult to determine the appropriate management as surgical options vary depending on disease extent. Many case studies have reported the benefits of MRI in detecting underlying DCIS.

We carried out a retrospective study on patients diagnosed with Paget’s diseases from 1st January 2012 to 31st March 2019 in 3 district general hospitals where MRI is not part of routine workup for Paget’s disease.

25 females (mean age 67) were diagnosed with Paget’s disease. Majority presented with nipple-areolar skin changes (21/25). All patients except one were assessed with mammogram and ultrasound. 5 patients had preoperative MRI. 22/25 patients were managed surgically (15 wide local excision; 7 mastectomies).Paget's disease with underlying DCIS identified on 16/22 surgical specimens (73%). 3/16 patients with DCIS demonstrated invasive disease. There was low detection rate of DCIS on pre-operative conventional imaging with only 6/16 DCIS detected. Multifocal DCIS was diagnosed on MRI in one patient with resultant mastectomy. Another patient had mastectomy for DCIS which was shown to be more extensive on MRI than mammogram. However, 3 patients needed repeat surgeries due to margin involvement, disease recurrence and widespread distribution of Paget’s disease. None of them had been assessed with MRI.

Given the potential for MRI to more accurately detect and size concomitant pathology, we propose that all patients with Paget’s disease have pre-operative MRI to allow appropriate surgical management from the outset.

### 0029 A pictorial review of Breast Lymphoma

#### Sowmya Veerasuri, Rebecca Geach

##### Southmead Hospital, Bristol, United Kingdom

###### **Correspondence:** Sowmya Veerasuri

**Background:** Lymphoma of the breast is a rare malignancy due to the dearth of lymphatic tissue. Breast lymphomas are categorised as either-Primary Breast Lymphoma (PBL) or Secondary Breast Lymphoma (SBL) with the latter being more common. Lymphomas of the breast in the absence of extramammary disease are referred to as PBL and have a worse prognosis.

Considering the infrequency of the disease and associated poor prognosis, it is important to understand imaging characteristics that may help in the detection and diagnosis. We present a pictorial review of breast lymphomas diagnosed at our unit.

**Methods:** A retrospective review of images from a prospectively maintained database of all patients with a histologically proven lymphoma of the breast between October 2016 and February 2019 was performed. Ultrasound, mammography, CT and PETCT images were reviewed.

**Results:** 20 patients with breast lymphoma were identified with a mean age of 69 years (range 28 – 87 years). Most consistent findings on imaging were an ill-defined mass/opacity. Architectural distortion, calcification and spiculation were less common. However, there were no specific ultrasound or mammographic features to predict breast lymphoma and diagnosis was confirmed on histopathology.

**Conclusion:** Given the overlap with other malignancies, breast lymphomas are challenging to diagnose based on imaging alone. However, the presence of extramammary lymphoma should raise the suspicion of breast lymphoma.

### 0030 Review of a new referral pathway for incidental breast lesions detected on Cross-sectional imaging

#### Sowmya Veerasuri, Helen Massey

##### Southmead Hospital, Bristol, United Kingdom

###### **Correspondence:** Sowmya Veerasuri

Incidental breast lesions detected on non-breast imaging are not uncommon and increasing. We developed a referral pathway where-in breast incidentalomas are highlighted via a dedicated e-mail and reviewed by the breast radiology team. Following review, selected patients are then assessed at a breast one-stop clinic. We reviewed the effectiveness of this pathway and also evaluated the outcomes of these referrals.

A retrospective analysis of cases between 30/01/2019 and 20/06/2019 was performed. Cross-sectional imaging was reviewed by a breast radiologist and reference made to previous imaging including screening mammograms. The data collected included the number of referrals to and the outcomes of the clinic review.

29 patients were referred with incidental breast lesions; 26 were detected on CT, 2 on PETCT and 1 on MRI. Of the 29 patients, 8(27%) were deemed not to require any further assessment following breast radiology review and were discharged.

Of the 21 patients assessed in clinic, 12 had benign lesions and 6(21%) patients had malignant lesions. Some example images of both lesions are presented.

The new referral pathway was able to decrease the number of patients referred to 1 stop clinic following an incidental imaging detected lesion by a quarter.  This not only saves unnecessary anxiety and a trip to hospital for those patients but also frees up valuable 1-stop clinic appointments.

In line with previous studies, a fifth of incidental breast lesions proved malignant in this series (Incidental breast lesions detected on CT: what is their significance? BJR;Vol 83, Issue 987,P233-240 March 2010).

### 0034 MRI-Guided Breast Biopsy: A Series Review with Histologic Correlation

#### Michael Courtney, Sylvia O'Keeffe, Aoife Maguire, Aoife Doyle

##### St James's Hospital, Dublin, Ireland

###### **Correspondence:** Michael Courtney

**Background:** To evaluate the predictive value of imaging characteristics on Magnetic Resonance Imaging (MRI) in patients undergoing MR guided breast biopsy. This study evaluates imaging features on the diagnostic MRI and which are more predictive of malignancy.

**Methods:** A retrospective review was performed of the Picture Archive and Communication System (PACS) for MRI-guided vacuum needle biopsies between September 2015 and July 2019. Studies were reviewed and lesion descriptor, size, radiology score, indication for diagnostic MRI and histology results recorded.

**Results:** 52 cases were included. 45 were successfully biopsied with 4 lesions no longer demonstrating enhancement at the time of procedure and 3 technically inaccessible. 48% were performed in women with newly diagnosed breast cancer, the strongest predictor of malignancy: 17 ipsilateral/11 malignant and 8 contralateral/3 malignant (malignancy rate 56%). 46% were performed in high risk women on surveillance (malignancy rate 16.7%) and 6% in women with normal conventional imaging and suspicious symptoms (malignancy rate 33%). 19 (42%) lesions were malignant: 4 scored MRI3, 5 MRI4, 2 MRI5 and 8 MRI6. Non-mass like enhancement was the most frequent descriptor of both malignant (8/19, 42.1%) and benign lesions (11/26, 42.3%). Size and imaging descriptor (mass, focus, nodule) were not predictive of malignancy.

**Conclusion:** No imaging characteristic was identified as a significant predictor of malignancy. There should be a low threshold for proceeding to MRI-guided biopsy particularly in women with newly diagnosed breast cancer in the absence of definitive findings on conventional imaging. 

### 0035 The clinical utility of Diffusion Weighted Imaging (DWI) in breast MRI: a pictorial review

#### Lyn Jones^1^, Anjum Mahatma^1^, Jonathon Delve^2^, Alice Pocklington^1^, Helen Massey^1^, Rebecca Geach^1^, Katherine Klimczak^1^, Alexandra Valencia^1^

##### ^1^Bristol Breast Care Centre within North Bristol NHS Trust, Bristol, United Kingdom; ^2^Medical Physics Department of North Bristol NHS Trust, Bristol, United Kingdom

###### **Correspondence:** Lyn Jones

Diffusion weighted imaging (DWI) is a non-contrast, functional MR technique that essentially provides a pictorial map of tissue cellularity. Already considered an important imaging sequence in prostate and neurological MRI, DWI is now being recognised as having the potential for clinical utility in breast MRI by providing diagnostic quality images of breast cancer without the need for gadolinium containing contrast enhancement.

A pictorial review is presented of the various appearances of breast carcinoma on DWI. It includes imaging with 1.5T and 3T and presents DWI images at a range of b values and also corresponding apparent diffusion coefficient (ADC) maps acquired on Philips (Amsterdam, Netherlands) Ingenia MR scanners with a dStream Breast 7 channel coil.

The emphasis of this presentation is on clinical utility and highlights the additional value of DWI sequences for women with mammographically dense breasts including those who have marked background parenchymal enhancement. The particular benefit of this technique to locally stage women with pregnancy associated breast cancer is also illustrated.

Images are included to illustrate typical appearances of triple negative, HER2+ and hormone sensitive carcinomas including lobular carcinoma on DWI. In addition, there are examples of the effective delineation of response to neo-adjuvant chemotherapy obtained from using this technique. The presented cases include corresponding images from dynamic and/or subtracted dynamic sequences and other sequences and modalities, where appropriate, to aid illustration.

### 0037 Addressing the British Breast Radiology workforce crisis, a Credential for Breast Clinicians.

#### Zoe Goldthorpe^1, 2^

##### ^1^President ABC, London, United Kingdom ^2^Associate Specialist Breast Clinician, Taunton, United Kingdom

###### **Correspondence:** Zoe Goldthorpe

**Background:** With a high retirement rate and increasing service demand, many British breast units have a radiology crisis. Screening and symptomatic targets are consistently difficult to achieve. This project between the Royal College of Radiologists, Association of Breast Clinicians and Health Education England will increase the number of Breast Clinicians (non-Radiologist doctors working in breast diagnosis, trained in breast imaging), standardise their training and provide formal accreditation by means of a Credential.

**Methods:** The new curriculum, delivered over three years has been developed by a project board. It follows General Medical Council guidance on curricula and features 14 Capabilities in Practice (CiPs) each focusing on one key element of generic or breast specific medical practice. The generic CiPs mirror those within the curriculum for Clinical Radiology and the trainee must demonstrate the ability to work within the multi-disciplinary team, engage in evidence based practice and exhibit all the professional values of a senior autonomous doctor. The breast specific CiPs set out the training requirements in mammography, ultrasound and interventional procedures. Trainees must pass the FRCR part 1 physics examination, learn non-imaging skills in clinical examination, understand oncological practice and assess family history and genetic risk.

**Results:** The new curriculum provides an innovative opportunity for doctors to train in breast imaging, diagnosis and risk assessment. Assessment is both formative and summative through examination and workplace based assessments.

**Conclusion:** The Credential delivers a solution to the breast radiology workforce crisis facing the UK now, providing accredited doctors, within three years.

### 0038 Should breast density be an indication for breast pre-operative MRI in patients with a primary breast malignancy?

#### Charlotte Longman, Koza Asanda, Natasha Davendralingam, Amy Agahi, Linda Metaxa, Tamara Suaris

##### Barts Health NHS Trust, London, United Kingdom

###### **Correspondence:** Charlotte Longman

Most breast units follow strict guidelines regarding the use of MRI in primary breast malignancy. Some indications are widely accepted, however dense breast parenchymal pattern is often overlooked as a true indication. Our aim was to determine the number of patients in which MRI demonstrated further lesions undetected on conventional imaging, and if these rates were higher in women with dense breasts.

Records of all patients presenting to our unit 2017 with a primary breast malignancy who underwent MRI were analysed. Breast density graded on initial presenting mammogram. MRI reports reviewed to determine which studies led to further imaging and whether further pathology was identified.

In total, 434 primary breast cancers were diagnosed in our unit. Of these, 174 (40%) patients had an MRI as part of their diagnostic pathway. Following MRI, 63/174 (36%) patients required second look ultrasound. 21/63 subsequent studies were normal, patient therefore proceeded with surgery without MRI altering treatment plan.

In 42/63 patients (66.7%), further pathology was identified. 17/63 (27%) patients had further sites of malignancy. The majority of patients with further pathology identified on MRI had dense breasts (ACR 3 and 4); 12/17 (70.5%) of those with a further malignant lesion identified, and 20/25 (80%) of those with further benign lesion.

MRI is an extremely useful tool in the diagnostic pathway of patients for surgical planning. Although density is not currently a strict criteria for pre-treatment MRI, our results suggest that careful consideration/increased utilisation of MRI should be made in those with increased breast density.

### 0039 Does pre-operative breast MRI reduce re-operative rates in breast cancer patients undergoing wide local excision, or simply delay their surgery?

#### Charlotte Longman, Amy Agahi, Koza Asanda, Linda Metaxa, Natasha Davendralingam, Tamara Suaris

##### Barts Health NHS Trust, London, United Kingdom

###### **Correspondence:** Charlotte Longman

MRI is increasingly used alongside conventional imaging in the diagnosis of breast malignancy, and has been publicised as the most reliable imaging technique to measure tumour size. However, there are concerns that MRI can lead to increased costs, over investigation of benign lesions, and treatment delays. Our purpose was to compare re-operative rates following wide local excision (WLE) in patients with and without pre-operative MRI, and to assess whether the addition of a pre-operative MRI causes significant treatment delays.

Records obtained of all patients presenting in 2017 with a confirmed histological diagnosis of breast malignancy, highlighting those undergoing WLE. Correlation with surgical pathological specimen and re-operative rates for positive margins in those patients with and without pre-operative MRI. Time from first presentation to treatment recorded in all patients.

In 2017, there were 434 cancer diagnoses in our unit. Of these, 273 (62.9%) underwent WLE. 91/ 273 patients undergoing WLE (33.3%) had a pre-operative MRI. 49/273 (17.9%) patients who underwent WLE required re-operative intervention for positive margins. Of those requiring re-operation, 35/49 (71.4%) had not undergone pre-operative MRI imaging. Mean time to treatment from initial presentation was 58 days (without MRI), 62 days (with MRI).

Pre-operative MRI is extremely beneficial in patients undergoing conservation surgery. A marginal increase in diagnostic time may reduce the possibility of positive margins and re-operation. There are comprehensive guidelines regarding indications for MRI, however we suggest careful consideration and potential increased utilisation in the pre-operative setting for those considering conservation, in order to reduce re-operative rates.

### 0040 Metastatic breast cancer mimics – the radiological pitfalls in common lesions seen on CT.

#### Flora Daley^1^, Penny Moyle^2^

##### ^1^Cambridge University Hospitals, Cambridge, United Kingdom; ^2^Cambridge University Hosptials, Cambridge, United Kingdom

###### **Correspondence:** Flora Daley

**Background:** In patients with a diagnosis of breast cancer CT imaging is an important part of the diagnostic and follow up pathway. The presence of bone, visceral lesions or lymphadenopathy on CT raises the suspicion of metastatic disease. Although easy to presume lesions are metastatic in the context of a known cancer, the radiologist must understand the differential diagnosis of lesions that may mimic breast metastatic disease.

**Methods:** We present interesting cases of breast cancer with coincidental pathology mimicking metastatic disease on CT.

**Results:** Cases include: lobular cancer with a concurrent undiagnosed multi-systemic sarcoidosis, osteopoikalosis in a ductal breast cancer mimicking multiple bone metastasis, haemagiomas mimicking liver and splenic metastases and silica lymph nodes in a women with breast reconstructions mimicking metastatic mediastinal nodal disease. We also list other key benign pathologies that can mimic metastatic disease to complete the knowledge on this subject.

**Conclusion:** This poster will remind the radiologist that breast cancer metastasis mimics can occur and although diagnostic uncertainly is unwanted, incorrect labeling of a patient with metastatic disease or missing a treatable non malignant process can be as devastating for the patient.

### 0042 Women with Dense Breasts: A Survey About Breast Density & Adjunct Screening

#### Robert Dickens^1^, Jonathan Nash^2^, Angelique Beling^2^, Sally-Ann Gibbs^2^, Rebecca Church^2^, Amy Hurley Dugdale

##### ^1^Western Sussex Hospitals NHS Trust, Chichester, United Kingdom; ^2^Portsmouth Hospitals NHS Trust, Portsmouth, United Kingdom

###### **Correspondence:** Robert Dickens

Breast density is a subject of interest and contention. This study’s objective was to gauge UK breast radiologists’ opinions on supplementary screening for women with dense breasts.

Anonymous survey deployed to members of the British Society of Breast Radiology.

123 responders of which: 84% document breast density; 8% have access to breast density software; 53% were aware of breast density associated increased relative risk of breast cancer. The design of the questionnaire allowed questions 5&6 to be skipped. It was assumed the 48/123 (39%) of responders who skipped these questions would not recommend supplementary screening. 75 clinicians did respond to questions 5&6. Of these 61 (81%) would offer supplementary screening for their patients, both NHS and private. 42% favoured digital breast tomosynthesis, 27% MRI, 20% breast ultrasound, 6% contrast enhanced mammography and 5% automated breast ultrasound. 59% expressed concern regarding false positives. 85/123 (69%) of responders believe we should be informing ladies of the reduced sensitivity of mammograms in the context of dense breasts. 36% believe we should be informing ladies of their heightened risk of malignancy. 28% expressed we should not be informing ladies of either of the latter two facts.

This survey demonstrates the variety of opinions on the controversial yet pressing issue of screening in the context of dense breasts. Availability of resources for reproducibly quantifying breast density is apparently poor. Attitudes expressed by responders hint at fears around limited breast imaging resources nationally. Further work is required to inform unified recommendations for women with dense breasts.

### 0044 Breast ultrasound elastography using size-ratio strain measurement and acoustic radiation force impulse elastographic techniques - which is better?

#### Llewellyn Sim, Tammy Moey, Lester Leong

##### Department of Diagnostic Radiology, Singapore General Hospital, Singapore, Singapore

###### **Correspondence:** Llewellyn Sim

**Objectives:** To evaluate and compare the diagnostic performance of breast elastography utilizing size ratio (SR) strain measurement and two acoustic radiation force impulse (ARFI) methods in the sonographic assessment of breast lesions.

**Materials and Methods:** From Oct 2016 to Jan 2018, 46 women with 50 breast lesions undergoing ultrasound-guided biopsy at our breast imaging centre were evaluated with ultrasound elastography prior to the biopsy. Each breast lesion was assessed independently by 2 different breast radiologists using SR on strain elastography, ARFI colour score (CS) and internal shear wave velocity (SWV) propagated by ARFI. Validation of radiological diagnosis was by histopathology. The sensitivity, specificity, positive predictive value (PPV), negative predictive value (NPV) and accuracy of each elastographic techinque were compared using Fisher's exact test.

**Results:** The mean age of the women was 50 years (range 29–79 years). Of the 50 breast lesions biopsied, 13 (26%) were malignant and 37 (74%) were benign. The sensitivity, specificity, accuracy and NPV of SR strain elastography were 100%, 73.0% and 80% and 100% respectively. The sensitivity, specificity, accuracy and NPV of CS using ARFI were 84.6%, 81.1%, 82% and 93.8% respectively. The sensitivity, specificity, accuracy and NPV of SWV using ARFI were 92.3%, 75.7%, 80% and 96.6% respectively. There was no statistical difference in the sensitivity, specificity and accuracy among the 3 elastographic techniques.

**Conclusion:** The diagnostic performance of the relatively newer ARFI elastography techniques were equivalent to the more established SR strain elastography.

### 0046 Recall rates in high risk women undergoing surveillance Breast MRI

#### Eman hafez^1^, Munir Mudassara^2^

##### ^1^Eman Hafez , Newcastle Upon Tyne , United Kingdom; ^2^Mudassar Munir , Newcastle Upon Tyne , United Kingdom

###### **Correspondence:** Eman hafez

**Background:** High risk women undergo breast MRI for surveillance as per NICE guidelines. MRI there is a risk of false positive resulting in increased recall rates with huge financial and also psychological impact on the patient causing anxiety. NHS breast screening guidelines has suggested a maximum recall of 7%.

**Aims:** Determine the recall rate in high-risk patients fromJan2016 till Dec2018. Comparing biopsy and cancer detection rate to results from 2008-2013.

**Standards:** NHSBSP guidelines suggest maximum recall rate of 10% with an expected recall rate of 7%.Our target was to improve the recall and cancer detection rate in these patients from our previous data of 9.2% recall rate.

**Material and methods:** Retrospective study of high risk MRI patients for breast cancer surveillance. Findings were graded, second look ultrasound and subsequent biopsy results followed.

**Results and recommendations:** 477 MRIs from 2008 to 2013 with recalls of 44.Cancer Detection of 2.1% and Recall Rate of 9.2% then re-audit.

Results re-audit:447 patients 92.2% were returned to screening and 7.8% were recalled.14.4% of those had normal US and 85.6% had biopsies performed following ultrasound.66.6% were B1/B2,13.% were B3 lesions and 20% were cancers.

**Conclusion:** Our recall rate was 7.8%, which shows improvement from last recall rate of 9.2% and is well below the maximum recall rate of 10%.Our biopsy rate was 85.6% with overall increased cancer detection rate of 20% from 2.1% of last time implying better cancer detection. Second read of MRI, consistent technique and close liaison with genetics team has helped greatly to improve service standards for this subset of patients.

### 0048 Oncoplastic breast conserving surgery: Challenges, complexities and pitfalls in pre-operative wire localisation.

#### Faisal Majid, Humaira Khan, Louise Tromans

##### SWBH NHS TRUST, Birmingham, United Kingdom

###### **Correspondence:** Faisal Majid

Newer breast oncoplastic conservation surgery such as pedicle perforator flap techniques (LICAP, MICAP) allow resection of large areas of DCIS or multifocal/multi-centric disease whilst maintaining good cosmetic outcomes. However, the perforator vessel to be used usually lies within the area requiring localisation. The focus is shifted from inserting the wire via the shortest route to not inserting the wire from the site of the vessel to ensure there is no damage.

We present some of our most challenging cases as well as how our service has evolved to accommodate these increasingly complex localisations.

The procedure will usually need to be stereotactic, but skin marking is ultrasound guided, thus increasing procedure time. The length of breast traversed is usually significantly greater, increasing the likelihood of wire migration and complications.

Once bracketed, surgeons will require multiple measurements to create a 3D rendering in their mind of the abnormal tissue.

Each case requires individual planning with the surgeon. At times in order to achieve good surgical margins, the bracketing is not for the lesion but to mark the limits of resection. Which makes targeting difficult and may mean the wire travels a much greater depth than would be ideal.

We have created a pro-forma for planning these cases. Any department where these procedures are being considered should also consider the added time taken to plan and execute accurate localisation. Newer techniques such as MAGSEED/Saviscout will help but still have limitations and cost implications.

### 0049 Breast Microcalcifications - Novel Biomarkers for Breast Tissue Pathology

#### Sarah Gosling^1^, Robert Scott^1^, Charlene Greenwood^2^, Pascaline Bouzy^3^, Jayakrupakar Nallala^3^, Iain Lyburn^4^, Nicholas Stone^3^, Keith Rogers^1^

##### ^1^Cranfield University, Shrivenham, United Kingdom; ^2^Keele University, Keele, United Kingdom; ^3^University of Exeter, Exeter, United Kingdom; ^4^Gloucestershire Hospitals NHS Foundation Trust , Cheltham, United Kingdom

###### **Correspondence:** Sarah Gosling

Microcalcifications are a key feature of mammography. Calcium phosphate (hydroxyapatite) microcalcifications are associated with both benign and malignant breast tissue and spectroscopic analysis has previously shown microstructure and chemistry variation between different tissue pathologies. It is well documented that the surrounding tissue microenvironment differs between normal and cancerous cells, notably pH and bicarbonate concentration. In addition, environmental factors can impact the formation mechanisms, and therefore the chemistry, of hydroxyapatite microcalcifications. This study hypothesises that the tissue microenvironment governs the formation mechanisms of hydroxyapatite crystals in breast tissue.

A total of 55 breast calcifications incorporating 3 tissue pathologies (benign – B2, ductal carcinoma *in-situ* – B5a and invasive – B5b) from archive formalin-fixed paraffin-embedded core needle breast biopsies were analysed using X-ray diffraction. The average distance between lattice discontinuities (coherence length) was determined from 538 diffractograms and crystallite size and non-uniform strain calculated using Williamson-Hall analysis.

Both crystallite size and non-uniform strain were found to increase with malignancy. An increase in crystallite size can be explained by a decreasing carbonate content with increasing malignancy. However, the increase in non-uniform strain is unexpected, but can be rationalised by differential substitution of carbonate into the hydroxyapatite lattice, controlled by the tissue microenvironment. An acidic extracellular pH is a characteristic feature of cancer cells. Our findings support a new hypothesis that pH and bicarbonate concentration can influence hydroxyapatite formation mechanisms through acidic precursors leading to differential carbonate substitutions. These cause measurable changes to the crystal microstructure, which act as novel biomarkers for breast malignancy.

### 0050 NBIA Breast Radiology Fellowship Programme - Update

#### Soujanya Gadde^1, 2^, Mary Wilson^1, 2^, Megan Bydder^1, 2^, Caroline Parkin^1, 2^, Paula Stavrinos^2^

##### ^1^Manchester Foundation Trust, Manchester, United Kingdom; ^2^National Breast Imaging Academy, Manchester, United Kingdom

###### **Correspondence:** Mary Wilson; Megan Bydder

NBIA Fellowship Programme has been developed to raise the profile of breast imaging, establish a gold standard post-CCT level breast radiology training programme and address the shortages in breast radiology work force by creating new routes of entry into the sub speciality. This 3 year rolling programme was developed in conjunction with HEE, who agreed to part fund the process.

A national fellowship model similar to Trans Interface Group(TIG) fellowships was developed. Working group with representation from the RCR, BSBR and several breast training units was set up. Eligibility criteria for centres to host a fellow were drawn. NHS Trusts from England were invited to apply by meeting eligibility criteria, demonstrating additional training capacity and securing the remainder of fellowship funding. A template business case was provided to assist with this. Entry criteria, curriculum, assessment and exit criteria for Fellowship were developed. Entry routes for international candidates were established. Posts advertised on NHS jobs and promoted via social media. Recruitment process was supported by South West deanery.

Fourteen eligible Trusts were approved. Fifteen fellowship applications received, ten interviewed and six fellows appointed for 2019 intake (three UK and three international). Candidates were matched to host centres based on their interview scores and centre preference. All candidates accepted offers.

There is demand for NBIA fellowships, both from breast training units and prospective fellows. Experience gained so far to help resolve teething issues, strengthen recruitment drive and attract a bigger number of high-calibre national and international candidates for next year's intake.

### 0051 Implementation of a Protocolisation and Triaging System to Streamline Breast Multidisciplinary Meetings and Improve Departmental Efficiency

#### Charlotte Longman, Virginia Wolstenholme, Jennifer Hu, Linda Metaxa, Jacky Jones, Tamara Suaris

##### Barts Health NHS Trust, London, United Kingdom

###### **Correspondence:** Charlotte Longman

Increased numbers of cancer diagnoses and complexity of cases place huge demands on multidisciplinary meetings (MDT). Prior to September 2018, we held a once weekly breast MDT involving 3 hospitals within the Trust, with 30-50 clinicians in attendance. A mean number of 89 cases were discussed weekly, averaging 4 hours in duration. Options to reduce list length would benefit a considerable number of people, particularly radiologists preparing for the meeting, at a time when demands on radiology are growing exponentially.

In September 2018, a “triage/ protocolisation” MDT was established. Two days prior to the main meeting the patient list was reviewed by a smaller MDT group involving 3 Consultants (Radiologist, Oncologist and Surgeon), a breast care nurse and MDT coordinator. Standard protocols were devised for patients in which a diagnostic/ treatment pathway could be easily established. These included T1a/b NO, T4d, disease recurrence and metastatic disease.  These pathways could be implemented at triage MDM saving main MDM discussion.

Following implementation, the main MDM list reduced significantly (mean 89 cases to 57 cases). This reduced both preparation time and MDM attendance time.  In 24% of pre-operative patients, a treatment plan was established during the triage meeting. 62 day performance improved, followed by 2 months of 100% compliancy (not previously achieved since November 2017).

The implementation has received extremely positive feedback with a demonstrable reduction in MDM preparation and attendance time, and measurable increase in departmental efficiencies.  We would highly recommend a protocolisation/ triaging system to other Trusts.

### 0057 Do all incidental breast lesions detected on CT need a one stop breast clinic appointment?

#### Lekha Potti, Wee Ching Ngu

##### North Cumbria University Hospitals NHS Trust , Carlisle, United Kingdom

###### **Correspondence:** Lekha Potti

The exponential rise in utilisation of CT imaging has led to an increase in detection of incidental breast lesions. These are usually directly referred to one stop breast clinic (OSBC) in many units. This can increase the workload of the already stretched breast services. We established a pathway to reduce unnecessary OSBC attendance and audited the results.

As per the pathway the CT radiologist would send a copy of the report to the Breast Radiologist. After reviewing the previous breast imaging, the Breast Radiologist would decide whether the patient needs referral to OSBC.

We aimed to determine if the established pathway reduced unnecessary OSBC attendance.

We collected cases of CT breast incidentalomas sent to the breast unit over a one year period (September 2016-17). Cases were collected via CT reports sent to breast radiologist, cases seen at OSBC and via Breast MDT.

22 cases were reviewed by the breast radiologist of which 13 (59%) did not need referral to OSBC as a result of the review. Of the 9 cases that were referred to OSBC, four were proven malignant, two proven benign, two patients died due to co-morbidities and one did not attend.

Our pathway avoided unnecessary OSBC appointments in two thirds of patients with CT breast incidentalomas. This enabled better utilisation of resources and reduced patient inconvenience.

### 0058 Pseudoangiomatous stromal hyperplasia (PASH) of the breast: a pictorial review of cases presenting to our unit in the last 4 years

#### Anju Nandhra, Naveed Altaf, Kaushik Dasgupta, Aninda Saha, Anuradha Anand

##### North Tees Hospital, Stockton, United Kingdom

###### **Correspondence:** Anuradha Anand

**Background:** First described by Vuitch et al in 1986, Pseudoangiomatous stromal hyperplasia of the breast (PASH) is a benign stromal lesion containing complex anastomosing channels lined by slender spindle cells. Though found incidentally in approximately 23% of breast biopsies, it often presents as a painless lump. We present the clinical, imaging and pathological findings of cases presenting to our Breast Unit in the last 4 years.

**Methods:** 28 cases were identified through the hospital's pathology database. Clinicopathological and imaging features were reviewed using our breast PACS and RIS.

**Results:** Average age of presentation was 49 years. 54% of cases were screen-detected; the remaining symptomatic.

On Ultrasound, 12 cases were well-defined solid lesions, 6 well-defined with mixed solid-cystic areas or with the appearance of fibrocystic disease, 2 demonstrated glandular tissue only and 1 had normal US features. Importantly 7(25%) demonstrated irregular or ill-defined masses. Of 25/28 cases where mammograms were performed, 3 were normal, 7 demonstrated asymmetric breast tissue, 9 well-defined masses,  3 partly well defined masses and 2(8%) were suspicious with ill-defined, irregular masses. 1 demonstrated parenchymal distortion.

2 cases presented as enhancing masses with type 1 enhancement curves on screening MRIs.

Histologically, PASH with cytological atypia may mimic angiosarcoma and other sarcomas.

**Conclusion:** In concordance with published literature, our cases of PASH often mimicked fibroademonata but also presented as glandular asymmetery, solid-cystic lesions and less often as suspicious ill-defined masses. PASH also formed enhancing masses on MRI; this is noteworthy for centres performing screening MRIs for high risk groups.

### 0060 Screening in the Over 70s: Informing the Debate with National Data

#### Sarah Savaridas^1^, John Quinn^2^, Andrew Evans^1^

##### ^1^University of Dundee, Dundee, United Kingdom; ^2^NHS National Services Scotland , Glasgow, United Kingdom

###### **Correspondence:** Sarah Savaridas

The risks and benefits of screening women >70yrs are unclear. This retrospective National study seeks to provide evidence to help older women make informed choices.

Aggregate Scottish screening data (2010-2015) was interrogated. Data was categorised by age, recall rates, biopsy rates, and tumour characteristics were recorded. Linked Cancer Registry data (2009-2013) and death records generated breast cancer mortality data. Data was recorded according to age at diagnosis with minimum 5yr follow-up. Cause of death was categorised as ‘primary breast cancer death’ or ‘non-primary breast cancer death’.

47235 women aged >70yrs attended screening, 5% were referred to assessment. Benign biopsy rate was 10%. Cancer detection rate was 14/1000. 29% of cancers were high grade (invasive/DCIS). One high-grade cancer will be detected for 248 women screened; but there will be 2.5 intermediate/low grade cancers and 1.2 benign biopsies. Between 2009-2013; 22013 Scottish women were diagnosed with breast cancer (screening and symptomatic). Although the majority of deaths, (4115/6697) in the follow-up period, were in those ≥70 years at diagnosis, the proportion of death due to breast cancer steadily decreases with age (*p*<0.0001). This trend remains significant for symptomatic women. There is a 2-fold decrease in proportion of deaths due to breast cancer between those aged 50-54years (0.87) and those aged 80-84years at diagnosis (0.43).

Benefits of screening women >70yrs may be out-weighed by the risks as they are more likely to die of other causes. This information should be available to women when deciding whether to be screened.

### 0063 Cancer detection in subcategories of NHSBSP High Risk Breast Cancer Screening

#### Maria Javed^1, 2^, William Teh^1, 2^

##### ^1^North London Breast Screening Service, London, United Kingdom; ^2^Northwick Park Hospital, London North West University Healthcare NHS Trust, London, United Kingdom

###### **Correspondence:** Maria Javed

NHSBSP high risk screening programme (HRSP) for family history and supradiaphragmatic irradiation (SRT) commenced in April 2013. The outcomes, cancer detection rates (CDR), and modality of detection in our institution were reviewed.

We retrospectively reviewed HRSP breast cancers between April 2013-2018. High risk category, diagnosis, lesion characteristics, imaging modality of cancer detection, biopsy method, and surgical histology were recorded.

There were 28 cancers (27/1160 screened) consisting of 19 invasive and 9 non-invasive cancers. Only 2 patients required MRI biopsy.

CDR was highest in BRCA1 women (42/1000; 13/313). All were invasive cancers. Cancers presented mainly as masses (92%; 12/13), with microcalcifications in only 1 patient. MRI had 100 % (9/9) detection rate. 6 had MRI and mammograms, with 4 mammogram occult cancers (67%).

There were 7 BRCA2 cancers consisting of 3 invasive and 4 DCIS (CDR 23/1000;7/307). Four were microcalcifications and 3 masses. There was 1 MRI detected mammographic occult cancer with a further 2 MRI detected malignant masses.

SRT had a CDR of 37.5/1000 (6/160) presenting as 4 masses and 2 microcalcification (4 invasive and 2 DCIS). Mammogram detected 4 and MRI detected 2 cancers.

There was only 1 Equivalent risk cancer (DCIS; CDR 16/1000, 1/63).

In summary, our study demonstrated that BRCA 1 carriers have the highest CDR, presenting mostly as masses, best detected on MRI, and frequently mammogram occult. BRCA 2 and SRT have a high rate of associated cancers, detected as microcalcification or masses, with a large proportion detected on mammograms. MRI biopsy was infrequently required.

### 0065 Is triple assessment of a breast lump always required or can we safely avoid core biopsies in the under 30’s with benign imaging?

#### Louise Merker, Jennifer Warner, Georgina Devenish, Diana Dalgliesh

##### Royal United Hospitals, Bath, United Kingdom

###### **Correspondence:** Diana Dalgliesh

Core biopsy is part of triple assessment. It allows for histological diagnosis leading to patient reassurance or malignant diagnosis, and guides further treatment. Complications are rare but it requires additional time in increasingly busy clinics. Malignancy is less likely to occur in younger patients. Our aim was to review this demographic and see if we could rely on benign imaging alone, thereby avoiding an unnecessary invasive procedure.

Patients aged 25 – 30 years who underwent a breast or axillary core biopsy in an 18-month period (Jan 1^st^ 2017 – June 30^th^ 2018) were included. Imaging was reviewed regarding radiological grading, specific ultrasound features and histological outcomes. Data was then compared to a 10-year review of all patients aged 25 – 30 years who had been diagnosed with breast cancer.

56 patients underwent biopsy. The majority, 66%, represented fibroadenomas. However, 3 were malignant, all with U4/U5 imaging. A single other case had U4 imaging but benign biopsy, which was felt to have been over called on retrospective review of the imaging. Within the 10-year review 19 patients in this age group had been diagnosed with a malignancy. 3/19 ultrasounds were graded as U3, all the others were U4 or above.

0.5% of all breast cancers diagnosed in our department in the last 10 years were in the 25 – 30 year group. If we had changed our local protocol to biopsy only U3 or above we would have captured all malignant lesions and safely prevented over two-thirds of unnecessary benign biopsies.

### 0066 Stereotactic Biopsy of Ultrasound occult lesions: 2 year review from a single screening centre

#### Megan Lewis, Nest Evans

##### Breast Test Wales, Cardiff, United Kingdom

###### **Correspondence:** Megan Lewis

**Background:** Stereotactic core biopsy is commonly used for sampling ultrasound occult lesions, being the mainstay for sampling of microcalcification. Some of the masses / asymmetric densities called back from screening mammography, while persistent on extra mammographic views, are not demonstrated on ultrasound. These then undergo stereotactic sampling.

**Aim:** To review pathology results of ultrasound occult masses and identify features that are predictive of the final pathological outcome.

**Method:** A retrospective review of 2 years of patients undergoing stereotactic biopsy for a mass lesion at our screening centre was undertaken. The records for these patients were reviewed to identify which patients had a normal ultrasound prior to stereotactic biopsy. The size and mammographic characteristics of the mass were compared to the final pathology results.

**Results:** 90 patients underwent stereotactic biopsy over the two year period for ultrasound occult lesions. The age range of these patients was 46-83. 21% of patients had a malignant result at final pathology.

21 patients were from the prevalent round, all of whom had benign pathology. Fibroadenomas accounted for 57% of the mammographic findings in these patients.

57 of the lesions were characterised as well defined, including both round and lobulated lesions. Only 3 of these were B5 on final pathology (5%).

**Conclusions:** 21% of stereotactic biopsies for ultrasound occult masses had malignant final pathology. This fell to 5% where the lesion was well defined. In the two years reviewed there were no ultrasound occult malignant masses in the prevalent round.

### 0067 Comparison of outcomes from opportunistic screening of the asymptomatic breast in women aged 40-50 presenting with unilateral breast symptoms with NHS breast screening (NHSBSP) outcomes

#### Harriet Standing^1^, Claire Ryan^2^, Bhavani Rengabashyam^3^

##### ^1^Mid Yorks NhS Trust, Wakefield, United Kingdom; ^2^St James’ Univeristy Hospital, LTHT, Leeds, United Kingdom; ^3^Leeds Breast Unit, Leeds Teaching Hospitals NHS Trust, Leeds, United Kingdom

###### **Correspondence:** Harriet Standing

Best practice diagnostic breast guidelines recommend bilateral mammography when women aged 40 and above with suspected breast cancers are referred by their GP through the two-week wait (2-WW) pathway. Our study examines the outcome of opportunistic screening of the asymptomatic breast in this age band when women present with unilateral breast symptoms and compare this with the NHSBSP data from Breast Screening Information System (BSIS).

Retrospective observational study. Women aged 40-50y, referred to the Leeds Breast unit under 2-WW GP referral pathway between January 2018 and December 2018 were identified from the hospital electronic patient records. Those with bilateral symptoms were excluded. Clinic letters, radiology and pathology reports were reviewed along with MDT discussions.

1935 women out of a total of 2227 referrals were imaged (86.8%).Out of the 85 cancers in 81 women, 8 (9.9%) were in the asymptomatic breast (4.1 cancers/ 1000 breasts screened). 66 women had 83 biopsies on the asymptomatic side. 68 benign, 4 B3 and 11 malignant (34.10 biopsies/1000 breasts screened). Asymptomatic cancers were small (63% </= 20mm), node negative (63%) and 25% were DCIS. In 2017/2018, NHSBSP detected 8.42 cancers/ 1000 women and performed 17.98 biopsies/1000 women screened for both breasts nationally.

Considering both breasts are screened in NHSBSP, this study from a single breast unit has identified that opportunistic screening of contralateral breast in 40-50y group presenting symptomatically detects similar number of cancers to NHSBSP but with a higher proportion of biopsies to achieve this. Multicentre studies would be helpful to compare and reflect.

### 0068 The Influence of Breast MRI on the Management of Invasive Lobular Cancer and its Accuracy in Correlation with Pathological Tumour Size – A 10-year Study

#### Hema Merai

##### Southend University Hospital, Southend, United Kingdom

###### **Correspondence:** Hema Merai

Invasive lobular cancer (ILC) constitutes 5-15% of all breast cancers and is frequently multicentric, multifocal and bilateral. Contrast-enhanced breast magnetic resonance imaging (MRI) improves the diagnostic accuracy in identifying these additional foci compared to conventional imaging, on which ILC is often occult. Consequently, surgical management changes in up to 50% of cases, in addition to potential overestimation of lesion size.

A retrospective observational study over a 10-year period of patients with a new histologically confirmed diagnosis of ILC, who had a preoperative contrast-enhanced breast MRI prior to surgical management. Lesion size on mammography, breast ultrasound and MRI was correlated with the post-operative histological size (which was considered as the gold standard) using the Bland-Altman analysis, with a ±5mm limit considered clinically concordance. Additional lesions identified on MRI were also analysed.

Between September 2007 and September 2017, 316 patients underwent breast MRI to exclude further disease after biopsy-proven ILC. 222 patients were included in this study. 113 additional lesions were identified on MRI in 77 patients (34.7%). Second-look ultrasound-guided biopsy was performed in 57.3% of the suspicious MRI lesions, of which 61.0% confirmed additional malignancy, thereby justifying any potential changes in surgical management.

With second-look ultrasound to prove the histology of additional foci found on MRI, this study provides supporting evidence for the targeted use of preoperative breast MRI in patients with ILC to optimise definitive surgical management by significantly correlating the size with final histology results and demonstrating low false positive rates.

### 0073 A clinical audit into the upgrade rates of breast lesions during second-line vacuum assisted core biopsies at a regional breast centre

#### Priyanka Nageswaran, Trupti Kulkarni

##### The Nightingale Centre, Manchester, United Kingdom

###### **Correspondence:** Priyanka Nageswaran

**Background:** Histologically, B3 lesions are classified as having “uncertain malignant potential” and are further categorised into various subtypes; each carrying a different risk of upgrade to pre-cancer or cancer tissue. Current guidelines from the National Health Service Breast Screening Programme (NHSBSP) recommend further sampling of B3 lesions.

**Aim:** To determine the histological concordance and upgrade rates of tissue specimens taken from breast lesions diagnosed initially by needle core biopsy (NCB) in comparison to second-line vacuum-assisted core biopsy (VACB) and demonstrate the impact of upgrade rates on patient outcomes. To further conclude whether current VACB techniques are favourable for clinical practice and patient management.

**Method:** A retrospective evaluation study in a three-year time period of patients (n=337) presenting for a second-line VACB after an initial NCB was carried out at a regional breast centre in a teaching hospital in South Manchester. Histological and radiological reports were reviewed.

**Results:** When comparing the histological concordance, there was no change in pathology in 40% of the patient cases. 21.4% of histological reports were upgraded to pre-cancer or cancer at second-line VACB. Surgical excision was further recommended by MDT in 5% of cases for definitive diagnostic purposes. All the patients diagnosed with malignant lesions on second-line VACB underwent definitive surgery without further diagnostic procedures.

**Conclusion**: The use of VACB in current practice has significantly decreased the number of patients having a diagnostic excision biopsy. The upgrade of breast lesions on second-line VACB has allowed patients to undergo definitive management with minimally invasive procedures.

### 0075 The ‘Breast Lump’ that is not in the Breast

#### Nandita Goel, Fiona Singh

##### WWL NHS Trust, Wigan, Manchester, United Kingdom

###### **Correspondence:** Nandita Goel

Occasionally we encounter ‘breast lumps’ presenting at the symptomatic clinic that are not arising from the breast. Lesions arising from deeper in the chest wall can clinically feel like a breast lump and are therefore referred to a symptomatic breast clinic.

We have recently encountered 8 such cases at our institution and we present their imaging findings, histology and discuss management. Diagnoses included benign spindle cell tumour, desmoid type fibromatosis, inflammatory ‘mass’ (previous sternotomy), plasmacytoma, granular cell tumour, simple and atypical lipomas and a low grade MPNST (malignant peripheral nerve sheath tumour).

All lesions underwent ultrasound, the majority of these lesions were not in the mammographic field of view. MRI is a useful investigation to assess the anatomical location, characterise the tissue composition of the lesion and assess vascularity/enhancement.

Pre-operative histology was obtained for some of these lesions by US-guided core biopsy in our breast unit. In some cases, following MDT discussion, it was decided that referral to a specialist soft tissue sarcoma centre was the best option as there is a potential risk of biopsy tract seeding/contamination in cases of malignant soft tissue sarcoma. NICE recommends biopsy of suspected soft tissue sarcomas in specialist centres. Histology was not straightforward and several of these cases had external second opinions which altered the final histological diagnosis.

The ‘breast lump’ that is not arising from the breast can be a challenging scenario for breast radiologists, pathologists and surgeons as we are diagnosing and managing non-breast lesions outside our comfort zone.

### 0076 Plymouth 3 year B3 lesion upgrade rate and national guidance compliance audit using stereotactic 10 gauge vacuum assisted biopsy as first-line.

#### Nicholas Carter^1^, Madalina Drumea^2^, Chong Yew Ng^2^, Karen Paisley^2^

##### ^1^University Hospitals Plymouth NHS Trust, Plymouth, United Kingdom; ^2^University Hospital Plymouth NHS Trust, Plymouth, United Kingdom

###### **Correspondence:** Nicholas Carter

**Background:** B3 Breast lesions have uncertain malignant potential. Due to the risk of coexistent malignancy surgical or vacuum assisted excision is usually recommended.

Increasingly there is a push towards conservative management preventing over-treatment, yet patients with B3 lesions, not proven cancer, are still undergoing surgical excision.

This audit compares our practice with the national B3 management guidelines. Our centre performs first line 10G vacuum biopsy for all stereotactic biopsies. We compare lesions diagnosed by 14G ultrasound biopsy to those diagnosed by 10G stereotactic biopsy to determine if first line 10G biopsy can minimise upgrade rates and/or reduce surgery.

**Method:** We reviewed the upgrade rates after excision for 174 patients with a B3 lesion over 3 years from April 2015 to April 2018 comparing our upgrade rates to the national average.

**Results:** Our upgrade rates are similar to the national rates. The difference in upgrade rates between 10G and 14G biopsies is marked in certain histological subtypes; for example, a 71.4% (5 of 7 patients) upgrade of AIDEP after 14G biopsy compared with only 9.1% (2 of 22 patients) after 10G biopsy. However, due to low numbers in each histological subtype we are not yet able to demonstrate a statistically significant difference.

**Conclusion:** With further data over the coming years, and an increasing appetite for conservative and minimally invasive treatment, there may be scope for a change of practice from 14G to 10G at first-line biopsy in order to minimise upgrade rates and the need for surgical intervention.

### 0078 National Health Service Breast Screening Programme (NHSBSP) clinical recalls: retrospective audit of patient outcomes in a UK breast screening centre

#### Sarrah El Tahir, Jo Swithenbank, Simon Lowes, Sally Athey, Preet Hamilton, Anju Nandhra, Jane Potterton, Alan Redman, Alice Leaver

##### Queen Elizabeth Hospital, Gateshead, United Kingdom

###### **Correspondence:** Alice Leaver

Women attending for screening mammograms as part of the NHSBSP are asked to report breast symptoms. Whilst perfoming screening mammograms, mammographers also asked to document any clinical signs they identify. These reported symptoms/signs can lead to recall on a clinical basis despite normal mammograms. Anecdotally, these recalls yield little pathology, potentially causing harm to women and utilising valuable clinic slots. This study evaluates cancer detection in this subset of women.

Retrospective audit was performed using electronic and paper records for women recalled to assessment by the NHSBSP for breast symptoms or signs between Jan 2014 and April 2015 despite normal two view digital mammography. Breast density was recorded in any cancers diagnosed. Descriptive statistics were performed.

Of 46178 women screened, 206 women were clinically recalled, 201 of whom had complete records available for analysis. Two women (2/201, 1%) were diagnosed with breast cancer at assessment. In both cases dense glandular tissue obscured the sonographically visible cancer. One of these women had a history of contralateral breast cancer (mastectomy). Thirty-four (34/199, 17%) clinically recalled women underwent intervention with benign results: 13 sets of core biopsies (13/199, 7%), 21 cyst aspirations (21/199, 11%). One cancer has subsequently been diagnosed in the reviewed population, contralateral to the site of clinical recall for symptoms.

Overall, these findings are consistent with the anecdotal understanding of a very low yield of breast cancer in this population, with arguable harm caused to a significant number of patients who were recalled.

### 0080 Difference in mode of presentation between invasive lobular carcinoma and invasive ductal carcinoma in a UK breast unit

#### Jo Swithenbank, Simon Lowes, Preet Hamilton, Alan Redman, Alice Leaver

##### Queen Elizabeth Hospital, Gateshead, United Kingdom

###### **Correspondence:** Alice Leaver

Anecdotally, invasive lobular carcinoma (ILC) is more difficult to perceive on mammogram than invasive ductal carcinoma (IDC). The UK National Health Service Breast Screening Programme (NHSBSP) aims to detect breast carcinoma of all types mammographically. This study investigates the proportion of ILC and IDC detected in our Trust through the screening programme compared to our symptomatic breast service.

Trust paper and electronic medical records were analysed for all patients diagnosed with IDC or ILC between January 2013 and December 2014. Descriptive statistics were performed.

656 IDCs and 110 ILCs were diagnosed in females over the study period. 41.8% (46/110) of ILCs were identified via the NHSBSP versus 50.6% (332/656) of IDCs. Remaining cancer detection was through the symptomatic breast service. Invasive carcinoma was mammographically occult in 8.2% of ILC (9 presentations to the symptomatic service, 9/110) and 4.6% of IDC (2 NHSBSP clinical recalls, 28 presentations to the symptomatic service: 30/656). The mean size of the largest focus of invasive disease at operative pathology was 28.2mm for ILC patients who had presented symptomatically, versus 21.6mm for ILC patients detected through the NHSBSP, 25mm for IDC patients who had presented symptomatically, versus 15.3mm for IDC patients detected through the NHSBSP.

A lower proportion of ILC than IDC diagnosed in our Trust is detected through screening compared to symptomatic presentation. NHSBSP two view mammography is, however, an effective tool for detection of both ILC and IDC, detecting cancers that are, on average, smaller than those diagnosed through the symptomatic service.

### 0081 The National Breast Screening Incident 2018: experience of Gateshead Breast Screening Service

#### Jane Potterton, Simon Lowes, Allison Wise, Janet Cumiskey

##### Queen Elizabeth Hospital, Gateshead, United Kingdom

###### **Correspondence:** Jane Potterton

Public Health England declared a national screening incident in May 2018 when it was understood that a large number of women had potentially missed their final invitation for breast screening between their 68^th^ and 71^st^ birthdays. Women thought to be affected were contacted: those up to age 72 were invited for screening while women aged 72 to 79 could request a screening appointment. This poster summarises the impact on one breast screening service.

Screening, administrative and pathology records were reviewed. Descriptive statistics performed.

697 women under 72 were sent a screening appointment, 601 women over 72 booked an appointment via the helpline. The service created an additional 38 clinics across 4 locations. Overall attendance for mammogram was 49% with a total of 1057 women screened.

Recall rate 3.7% (37 cases). 32 biopsies were performed in 25 patients. 19 cancers detected (17.9/1000 screened), 18 invasive, 1 DCIS. Of the invasive cancers 4 were Grade 1, 12 Grade 2 and 2 Grade 3. 2 cases had involved lymph nodes. Mean tumour size 18mm (range 3-60mm). All cancers underwent surgery, one following neoadjuvant chemotherapy. 3 women had pre-treatment MRI. 1 B3 lesion was referred for annual mammography.

Administrative staff received a substantial increase in telephone calls during incident. Radiographers reported more client questions. Core programme was maintained.

Overall the catch up exercise following the 2018 “incident” resulted in a burden on all aspects of our service. As expected for the age group, there was a high cancer detection rate.

### 0082 Preoperative MRI measurement of invasive breast cancer post-neoadjuvant chemotherapy (NAC): comparison with operative histology in a UK breast unit

#### Preet Hamilton, Simon Lowes, Alan Redman, Alice Leaver

##### Queen Elizabeth Hospital, Gateshead, United Kingdom

###### **Correspondence:** Preet Hamilton

Accurate interpretation of contrast enhanced breast MRI is required to evaluate response to NAC, to guide further patient management including surgery.

Our aim was to determine the relationship between invasive disease measurement at preoperative MRI and at final excision pathology.

MRI workstation records identified all patients who underwent evaluation of breast cancer response to NAC between October 2012 and June 2018; largest MRI residual tumour dimension recorded was compared with largest measurement documented at final operative pathology.

Patients were excluded if complete data was unavailable. Where both non-invasive and invasive disease was present then the largest invasive size was used. Regression analysis was performed.

Of 66 patients undergoing MRI for NAC, 26 were excluded. 25% (10/40) exhibited complete response on both MRI and surgical pathology, 15.5% (5/40) exhibited complete response on pathology but not MRI, and 10% (4/40) had complete response on MRI but residual disease on pathology. The longest dimension of residual invasive malignancy on MRI correlated with final histological tumour size (r2=68%, p=0.00001), but MRI tended to overestimate disease. There was no significant difference in MRI performance between tumour grades 2 and 3 (r2=0.73% grade 2, r2=0.70% grade 3) or ER status (negative r2=0.63, positive r2=0.66); correlation between MRI and pathology measurements was greater for HER2 negative disease (r2=0.76) than positive disease (r2=0.53).

In conclusion, although contrast enhanced breast MRI post NAC is an effective means of predicting size and presence of residual invasive disease at surgical excision, it has a tendency to overestimate disease.

### 0083 Consultant Radiographer in breast imaging: Entry into a growing role

#### Richard Morrell, Alice Leaver, Simon Lowes

##### Queen Elizabeth Hospital, Gateshead, United Kingdom

###### **Correspondence:** Alice Leaver

There is an established workforce crisis in UK radiology. Contributing factors include rising demand, retirement, an increasing numbers of vacant posts, and a shortfall in supply of radiologists. The most recent Workforce Census once again identified breast imaging as having the biggest shortage of radiologists, with a 9.3% unfilled vacancy rate.

To help maintain breast services, there has been a move to developing existing Allied Health Professionals (AHPs) into Consultant Radiographer roles. However, the route to Consultant Radiographer is not always clear, and for Trusts developing this role for the first time it can be a challenging process.

The role of Consultant AHP Practitioners was born from the Department of Health publication “Meeting the challenge: a strategy for allied health professions (2000)” and “The NHS plan”. Both documents proposed developing areas of responsibility for Consultant AHP Practitioners, defining the role as multidimensional positions encompassing the core functions of consultant practice: expert clinical practice; professional leadership and consultancy; practice and service development, research and evaluation; education and professional development.

We illustrate, from different staff perspectives, our experience of developing a Consultant Radiographer in breast imaging. We outline the potential different career routes into this role, including our example of recruiting from a non-mammographic background.

The principal challenge is a lack of a formally established training pathway within the UK. It should also be recognised that AHPs from a non-mammographic background have valuable transferable skills that can provide an alternative pathway to the role of a Consultant Radiographer.

### 0085 Surgical re-excision rates for patients undergoing breast-conserving surgery: comparison between invasive lobular carcinoma and invasive ductal carcinoma

#### Sarah Carpenter, Ishaana Munjal, Sai Chow, Preet Hamilton, Alan Redman, Simon Lowes, Alice Leaver

##### Queen Elizabeth Hospital, Gateshead, United Kingdom

###### **Correspondence:** Alice Leaver

Our Trust has recently demonstrated that invasive lobular carcinoma (ILC) is less accurately measured on preoperative imaging than invasive ductal carcinoma (IDC). To help assess the patient impact of preoperative disease measurements made on imaging, this study compares surgical re-excision rates for IDC with or without associated ductal carcinoma in situ (DCIS) and ILC with or without associated DCIS.

Retrospective audit was performed of all ductal and lobular cancers diagnosed and treated in our Trust between January 2013 and December 2014. Hospital electronic records were used to identify patients, their imaging, surgical procedures and pathology. Descriptive statistics were performed.

Full records were available for 528 patients who underwent breast conserving surgery for ILC (53), and IDC (473); there were also two cases of mixed ILC and IDC within the WLE specimen.

Rate of positive margins for IDC was 15% (74/479), for ILC was 17% (9/53), and for mixed was 50% (1/2). Of these, re-excision rates were IDC (57 %, 42/74), ILC (78%, 7/9) and 100% for mixed (1/1). The mean number of further re-excisions per patient who had re-excision was 1 for the ILC group, 1.1 (47procedures/42patients) for IDC, and 100% for mixed (1/1). Mastectomy was the final surgical procedure in that treatment episode for 4% (2/53) cases of ILC, 3% (16/473) cases of IDC, and 0% (0/2) cases of mixed IDC/ILC).

Overall, surgical re-excision rates were highest for ILC. These results emphasise the importance of preoperative imaging including measurements, and of developing more accurate ways to do this.

### 0086 A new breast localisation technique: use of the Hologic LOCalizer radiofrequency ID tag system in a UK breast unit

#### Alice Leaver, Sunil Amonkar, Rob Milligan, Jane Potterton, Preet Hamilton, Simon Lowes

##### Queen Elizabeth Hospital, Gateshead, United Kingdom

###### **Correspondence:** Simon Lowes

The Gateshead Health NHS Foundation Trust is the first in the UK to implement the Hologic LOCalizer radiofrequency ID (RFID) tag system to localise non-palpable breast cancers prior to surgery. The aim of this project was to assess the utility of this system in a UK breast unit.

The first tags were delivered in June 2019. Two NHS oncoplastic breast surgeons recruited all willing breast patients (from both screening and symptomatic presentations) who would otherwise have undergone traditional guidewire localisation. Prospective audit was performed on insertion, surgical excision, pathology, and any complications. Descriptive statistics applied.

39 tags were inserted into 36 female patients between 12 June and 21 August 2019, 1-26 days before their planned date of surgery. To date, 21 patients have had their lesions/tags surgically excised (23 tags in total). All tags were ultrasound-guided and included masses (invasive tumours and mass-forming DCIS), two post-stereotactic vacuum-assisted biopsy cavities, and two cases targeting of clips post neo-adjuvant chemotherapy. No significant procedural difficulty/complication was encountered with tag insertion, surgical localisation, or excision, with a short learning curve. One patient did, however, have a further incidental focus of malignancy identified and biopsied at the time of tag insertion necessitating additional guidewire localisation on the morning of surgery.

Pathology staff have encountered no difficulty with the tags, which are easily disposed of in clinical waste. So far all excised lesions have had negative margins, resulting in no re-excisions.

Initial results suggest the LOCalizer RFID system to be an effective alternative to guidewire localisation.

### 0087 Mammogram and ultrasound appearance of grade 3 breast cancers: a 2 year retrospective review

#### Sai Chow, Alice Leaver, Simon Lowes, Alan Redman, Preet Hamilton

##### Queen Elizabeth Hospital, Gateshead, United Kingdom

###### **Correspondence:** Alice Leaver

Early detection and treatment of grade 3 breast cancer is important to patient prognosis. This study retrospectively reviews mammogram and ultrasound appearances of grade 3 breast cancers diagnosed in our Trust over a 2-year period.

All patients (screening and symptomatic) with grade 3 breast cancer histology diagnosed between January 2013 to December 2014 were identified using hospital electronic records. Images were reviewed. NBSS descriptors were used to describe cancer appearance on mammogram and ultrasound. Occult lesions were noted. Descriptive statistics performed.

235 female patients were diagnosed with grade 3 breast cancer over the 2 year period, 72 screen-detected and 163 symptomatic. On mammogram, most cancers were ill-defined masses (73%, 171/235), 99% (168/171) of which had associated microcalcification (MCL). Grade 3 cancers rarely presented as an asymmetric density (8%, 18/235), MCL with no associated mass (7%, 16/235), clinical recall/mammogram occult (6%, 15/235), well-defined mass (6%, 14/235), architectural distortion (1%, 3/235), or lymph node abnormality (0%, 0/235).

On ultrasound, all grade 3 cancers were described either as an ill-defined mass 91% (215/235) or as a well-defined mass (9%, 20/235). Mean tumour size on surgical excision was 20.5mm for screen-detected cancers (range 6-80mm, median 16mm), and 31mm for symptomatic cancers (range 9-200mm, median 24mm).

In conclusion, grade 3 invasive breast cancers most commonly present on mammogram as an irregular mass with associated microcalcification, and as an ill-defined mas on ultrasound. It is notably rare for a grade 3 cancer to present as an architectural distortion or as an isolated lymph node abnormality.

### 0088 Subjective assessment of nodal cortical thickness by radiologists: impact upon prediction of lymph node metastases at surgery

#### Simon Lowes, Nikhil Birdi, Alan Redman, Alice Leaver

##### Queen Elizabeth Hospital, Gateshead, United Kingdom

###### **Correspondence:** Simon Lowes

One of many controversies surrounding managing the axilla in breast cancer is whether ultrasound can predict the presence and extent of nodal metastases pre-operatively, including establishing the optimum cortical thickness at which needle biopsy should be carried out. Our unit has a relatively low threshold of 2mm. We evaluated how having a fixed threshold influences our decision to biopsy and how our practice influences outcome.

Retrospective analysis was carried out for all patients with a B5 breast core biopsy between Jan 2013-Dec 2014. Ultrasound images/reports were assessed for cortical thickness measurements; pathology results were accessed for axillary surgery outcomes.

After exclusions, 801 patients with breast cancer had axillary ultrasound; 711 underwent axillary surgery, of whom 669 had a measurable cortical thickness (42 had rounded/replaced nodes).

Measured cortical thicknesses range was 0.5-14 mm overall (single thickest cortex per patient), with 49.8% measured at <2mm and 50.2% ≥2mm. 10% (67/669) of all cortices were measured at exactly 1.9mm, i.e. just under the biopsy threshold, and only 0.9% of cortices measured at the 2mm threshold itself. Just 3.3% of measurements fell into the range 2.0-2.3mm, suggesting some subjective bias regarding decision to biopsy.

Of those measured at 1.9 mm, 9% (6/67) had metastases at surgery, compared to 14% (5/36) measured within the 2-2.3mm range (in all cases ≤2 nodes positive), suggesting any subjective measurement/decision not to biopsy did not comprise outcome.

This reinforces that ultrasound assessment of the axilla remains subjective despite fixed biopsy thresholds, though at our current threshold it remains safe.

### 0091 What is the picture for papillomas and radial scars two years on from the implementation of new National Health Service breast screening programme (NHSBSP) B3 lesion guidance?

#### Juliet Morel, Bhavna Batohi, Cristian Monaco, Barbara Barbosa, Chirag Shah, Jane Goligher, Michael Michell, Clare Peacock, Rumana Rahim, Ali Sever, Rema Wasan, Shalini Wijesuriya, Keshthra Satchithananda

##### Kings College Hospital, London, United Kingdom

###### **Correspondence:** Bhavna Batohi

In 2016, guidance was released for the management of B3 lesions. Guidance for radial scars/complex sclerosing lesions (RS/CSL) and papillomas is to perform vacuum assisted excision (VAE) in lesions with no atypia shown on diagnostic biopsy and surgical excision when atypia is present. We have audited adherence to guidance and upgrade rates to malignancy.

Papillomas and RS/CSL diagnosed within screening and symptomatic women between 01/01/2017 to 30/01/2019 were identified using the pathology database. For each lesion, imaging features and type of initial biopsy was documented. The initial histology, including presence of atypia, and histology from VAE or surgical biopsy were recorded. Upgrade rates to malignancy were quantified.

148 lesions were identified. Nine were excluded due to synchronous cancer in the same breast, leaving 139 lesions. 90 lesions were masses, 35 distortions, 13 microcalcifications and 1 asymmetric density with size range of 2-80 mm.

There were 84 papillomas, 9 with atypia. 54/84 of these underwent VAE and 18 underwent surgical excision. 12 lesions, all without atypia, had no further sampling. Upgrade for papillomas with atypia was 33.3% (3/9) and without atypia 1.6% (1/63).

There were 55 RS/CSL, 4 with atypia. 46/55 of these underwent VAE and 5 underwent surgical excision. 4 lesions, all without atypia, had no further sampling.  Upgrade for RS/CSL with atypia was 25% (1/4) and without atypia 6.3% (3/47).

Guidance was well adhered to at our institution. The low upgrade rates of papillomas and RS/CSL without atypia supports the new guidance replacing surgical excisions with image-guided VAE.

### 0092 Microcalcification: what is the optimal mammographic assessment?

#### Rumana Rahim^1^, Rema Wasan^1^, Aris Vasilogiannakis^2^, Asif Iqbal^1^, Bhavna Batohi^1^, Juliet Morel^1^, Shalini Wijesuriya^1^, Michael Michell^1^, Clare Peacock^1^, Jane Goligher^1^, Keshthra Satchithananda^1^

##### ^1^Kings College Hospital, London, United Kingdom; ^2^Worcestershire Acute Hospitals, Worcester, United Kingdom

###### **Correspondence:** Bhavna Batohi

Microcalcification is a common finding. A standard workup may include a lateral projection of the whole breast and lateral and CC paddle magnification views. Some centres no longer use magnification views, relying on digital zoom. The use of digital breast tomosynthesis (DBT) varies.

Our centre evaluates microcalcification with co-registered 2D digital mammography (2DDM) and DBT in the lateral projection and lateral and CC paddle magnification compression views.

Retrospective review of 103 consecutive cases of histologically confirmed malignant microcalcification using 9G x-ray guided vacuum (VAB).  Mammographic views were read sequentially and scored M3-5. Additional soft tissue on DBT was recorded. Continuous zoom of up to x8 was used.

Lateral DBT and magnification views independently increased mammographic suspicion in comparison to 2DDM with statistical significance. 20.6% were scored M4/5 on 2DDM. This compared with 54.9% on DBT (p-value 0.0002), 49.5% with magnification views (p-value 0.0002) and 16.8% with lateral 2D (p-value 0.472).

37.5% of DBT cases demonstrated soft tissue that was not visible on the 2D mammograms increasing the suspicion level in those cases. 13 cases in the study showed an upgrade from VAB at final surgical pathology. 77% (10/13) of the upgrades had a soft tissue abnormality on DBT.

We recommend magnification views for the optimal assessment of microcalcification. Lateral DBT also improves reader confidence in predicting malignancy compared with 2DDM and may aid biopsy targeting. The 2D lateral mammogram had no benefit in this study; we propose synthetic 2D imaging with DBT could replace this.

### 0094 9 year retrospective study of Phyllodes tumours- A teaching Hospital experience

#### Aisha Naseer, Alexander Wilson, Nyla Khan, Philip Dilks, Shefali Dani

##### St Bartholomew's Hospital, Barts and The Royal London NHS Trust, London, United Kingdom

###### **Correspondence:** Nyla Khan

Phyllodes tumour or cystosarcoma phylloides are fast growing tumours arising from periductal stromal cells of the breast. Triple assessment has low sensitivity and poor diagnostic accuracy in differentiating them from benign lesions. No clear guidelines exist on subsequent management of phyllodes tumour.

Data obtained from electronic records: clinical, imaging, surgical and histology based for all patients with suspected phyllodes on any investigation dating back to 2010 (9 years).

An assessment of imaging features, initial core biopsy, final excision biopsy result, presence of clear surgical margins, the recommended follow up and whether this was implemented and presence of recurrence.

76 confirmed cases of phyllodes were sub-classified into benign (63%), borderline (23%), and malignant (14%) on excision biopsy. The lesion was appropriately graded in 61% on initial core biopsy. 78% were graded as Category 2/3 on UK 5 point imaging system; mean age was 36 years and lesion size 32mm. Follow up was documented in only 37 patients, predominantly with annual mammogram for 5 years. Recurrences were demonstrated in 5, of which 4 had unclear margins, and the following histological grades: benign (2), borderline (2) and malignant (1). The mean time for recurrence was 23.6 months. There were no distant metastases.

Imaging findings are not representative of subsequent histological grade. Whilst relatively few cases demonstrated recurrence, these were related to incomplete surgical excision and where follow up was implemented, illustrating the importance of this. Based on our experience, a management algorithm for the follow up of phyllodes tumours has been proposed.

### 0095 The growing fibroadenoma – To rebiopsy or not!!!

#### Sonali Shah, Nyla Khan, Tamara Suaris, Shefali Dani

##### St Bartholomew's Hospital, Barts and The Royal London NHS Trust, London, United Kingdom

###### **Correspondence:** Nyla Khan

Fibroadenomas (FA) are common biphasic benign fibroepithelial tumours composed of stromal and epithelial elements. The epithelial component of a FA can display the same pathologic aberrations as those of the breast. Natural history suggests that 25% of these lesions will get bigger. However, there are no definite guidelines concerning an acceptable growth rate of biopsy proven FA, when to safely follow up vs rebiopsy. The purpose of this study was to determine the incidence of malignancy in the growing fibroadenoma at our institute, to establish local rebiopsy guidelines.

A retrospective review of all biopsy proven B2 FA using RIS, PACS and pathology database was performed to identify any growing fibroadenoma over a 3 year period. B3 lesions at initial biopsy were excluded.  Maximum single dimension at T0 (time of initial core biopsy) and T1 (time at largest measurement) were recorded. Percent growth and length of time was recorded.

122 patients were identified over 3 years. The mean age was 32 yrs. Mean T0 was 18mm (range 3-60mm). Average interval growth was 62% with time to rebiopsy of 34 months (range 2mths – 8 years). 4 patients developed subsequent benign phylloides (3,2% incidence, all with % growth >30%) with no malignant transformation.

Literature suggests that FA’s may be safely followed up with a mean change in dimension of 20% over a 6 month period for all ages. FA’s may enlarge due to natural progression, possible development of epithelial malignancy or misdiagnosed phylloides. We recommend rebiopsy only if size increases > 30%.

### 0097 A breast unit’s experience of 15 cases of lesion localisation prior to surgery using radiofrequency (RF) seeds – a radiological, surgical and pathology review.

#### Vaishali Gada, Joan Butt

##### Wycombe Hospital, High Wycombe, United Kingdom

###### **Correspondence:** Joan Butt

Ten cases which would normally require wire localisation of lesions prior to surgery underwent radiofrequency seed (RF) placement.

Currently wires are placed on the day of surgery and this can result in a delay in patients being ready for surgery. The trial aimed to identify the benefits and drawbacks of the RF seed procedure. Ease of placement for radiology, both technically and for workload planning were recorded. Any comments from the surgical team with changes in knife to skin times and any changes in surgical margins were recorded.

The RF seeds have licence for 30 days implantation which the manufacturer is hopeful will be extended under licence. If the procedure had a positive response by all parties, there may be marked benefits for patients and for workload planning. The higher cost of the RF seed, applicator and intraoperative probe may make the procedure cost effective compared to the cost of a localisation wire if concurrent benefits could be identified.

All patients consented to the procedure. In this trial RF seeds were implanted on the day and the procedure was well tolerated by all patients.

RF seeds were implanted both under ultrasound and x-ray guidance. All cases had post insertion mammography in the lateral and cranio-caudal projection combined with ultrasound guided skin marking to indicate the depth and position of the RF seed before proceeding to surgery.

The poster will aim to identify the benefits and disadvantages of RF seed localisation.

### 0098 Audit of implementation of new guidelines in management of B3 lesions with Vacuum-Assisted Excision Biopsy at a District General Hospital (Princess Alexandra Hospital, Harlow, Essex)

#### Helen Newman^1^, Pauline Rajan^1^, Anthony Aylwin^1^, James Diss^2^, Seena Thomas^1^, Hema Jeyachandran, Preethi Gopinath

##### ^1^Epping breast unit, Epping, Essex, United Kingdom; ^2^PAH NHS Trust, Harlow, Essex, United Kingdom

###### **Correspondence:** Pauline Rajan

**Background:** New NHS Breast Screening Programme guidance recommends that surgical excision/biopsy for most screen-detected B3 lesions of uncertain histological malignant potential is not required and that Vacuum-Assisted Excision (VAE) should be performed for further characterisation.

**Method:** In this audit, following implementation of the new guidelines, 47 screen-detected B3 lesions at initial biopsy (31 core and 16 vacuum-assisted biopsies) at our screening unit were reviewed to assess NHS BSP B3 Guideline compliance.

**Results:** Following interrogation of NBSS, pathology and MDT outcomes, 27 cases (57.5%) were found to be compliant with the guidelines. Of the remaining 20, initial 10G vacuum-assisted biopsy completely excised the whole lesion in 7 cases, 6 had coexistent invasive disease that determined the subsequent management and 3 transferred their care elsewhere after the initial biopsy. In a further 4 cases, surgery was performed because the recommended VAE was not technically suitable (due to lesion size and/or location in the breast). Overall 23% of VAEs showed pathological upgrade (in all cases to DCIS) from the initial diagnosis; 54% of the excision biopsies resulted in no change to the lesion grading; and the lesion was downgraded in 23%.

**Conclusion:** This audit demonstrates reasonable compliance with the new NHS BSP B3 Guidelines, with all deviations from them justifiable and ratified at the Multi-Disciplinary Meeting. Indeed, since their introduction in 2016, surgical excision has only been performed according to guideline recommendations, or where VAE was not practically possible.

### 0100 A fresh look at CT staging for breast cancer: can we do better? Part II - analysis of more than 1000 patients

#### Natalia Roszkowski, Selina Lam, Michael Couzins, Rachel Oeppen

##### University Hospital Southampton, Southampton, United Kingdom

###### **Correspondence:** Selina Lam

Nationally there are differing approaches to staging for distant metastatic disease in breast cancer. This study aims to identify factors predictive of metastatic disease in order to refine current guidelines. A further year of data has been collected since previously presented.

Retrospective data were collected for all patients with new invasive breast cancer over a three-year period (January 2014 to January 2017) at our institution. Data were collated and analysed using Microsoft Excel and Regressit software. Positive predictive values and binomial logistic regression analysis were applied.

1377 patients with breast cancer were identified (mixed screening and symptomatic cohort), with complete data available for 1025. Staging CTs were performed for 323. Distant metastases were identified at presentation in 47 (4.6%). Thirty of the 47 metastatic cases met established criteria for staging (i.e. T4, recurrence, symptoms of distant metastases), leaving 17 cases potentially missed. Systematic multivariant analysis shows that, in addition to established staging criteria, tumour size 3cm or greater in combination with sonographically abnormal axillary nodes also predicts an increased rate of metastatic disease at presentation (PPV=18.8%, OR=4.831, p<0.005). Applying this additional criterion increases positive CT rate to 17.1% (previously 14.6% at our institution) while 95 fewer CTs are performed (29% fewer).

We propose that pre-treatment CT staging is warranted if breast tumour size is 3cm or greater with concurrent axillary node positivity on ultrasound, in addition to existing established criteria. This improves detection of distant metastatic disease and CT positivity rate, without an increase in the number of CTs performed.

### 0101 Abbreviated Breast MRI – a systematic review

#### Rebecca Geach^1^, Samantha Harding^1^, Andrea Marshall^2^, Janet Dunn^2^, Lyn Jones^1^

##### ^1^Bristol Breast Care Centre within North Bristol NHS Trust, Bristol, United Kingdom; ^2^Warwick Clinical Trials Unit, Warwick, United Kingdom

###### **Correspondence:** Rebecca Geach

Breast MRI is the most sensitive screening modality for breast cancer but its lengthy acquisition and reporting times result in a high unit cost that restricts its use. In the UK it is only used to screen women at the very highest risk of developing breast cancer (>30% lifetime risk).

Abbreviated breast MRI was designed with the long-term goal of increasing access to screening breast MRI. The concept was to limit the examination to include only the elements of the full protocol with the most useful diagnostic information whilst minimising time spent on the scanner by the screening client and radiology time to report the scan. Since Christiane Kuhl’s original proof of concept study was published in 2014, research groups from Europe, North America and the Far East have also investigated whether it is possible to design an abbreviated protocol that offers the accuracy of the full protocol breast MRI for cancer detection with the brevity of a mammogram’s acquisition and reporting times.

This talk explores the published evidence, and includes discussion of recent UK research into the potential for multi-professional reporting.

### 0102 Breast lymphoma – a pictorial review of its multimodality features with pathological correlation

#### Nyla Khan, Linda Metaxa, Valentina Sangiorgio, Simon Hallam, Tamara Suaris

##### St Bartholomew's Hospital, Barts and The Royal London NHS Trust, London, United Kingdom

###### **Correspondence:** Nyla Khan

To present and describe the diverse imaging features of primary and secondary breast lymphoma on mammography, ultrasound, computed tomography and magnetic resonance imaging. This remains a challenging entity to diagnose based on its non-specific imaging features, with careful scrutiny of patient history, clinical suspicion and histopathological correlation crucial in diagnosis and management.

Patients who had a definite pathological diagnosis of breast lymphoma were identified from a database. The imaging features of these cases across various modalities were reviewed.

Whist the majority of breast lymphoma cases demonstrate features overlapping those of breast tumors, key clinical and radiological features can assist in discriminating from other common benign and malignant lesions affecting the breast. The most common manifestation of breast lymphoma is presence of a solitary breast mass, enlargement of ipsilateral axillary lymph nodes and subcutaneous involvement. A lack of calcification and architectural distortion is noted. Breast implant-associated anaplastic large cell lymphoma is an entity that needs to be suspected in cases of new massive peri-implant fluid collections containing thick septations and solid detritus or development of masses related to the fibrous capsule.

Breast lymphoma is not commonly encountered. Our pictorial series highlights a variety of presentations across a range of imaging modalities which we hope will assist radiologists and clinicians in diagnosing this challenging and rare hematological neoplasm.

